# Boosting the Adhesivity of π-Conjugated Polymers by Embedding Platinum Acetylides towards High-Performance Thermoelectric Composites

**DOI:** 10.3390/polym11040593

**Published:** 2019-04-01

**Authors:** Tao Wan, Xiaojun Yin, Chengjun Pan, Danqing Liu, Xiaoyan Zhou, Chunmei Gao, Wai-Yeung Wong, Lei Wang

**Affiliations:** 1 Shenzhen Key Laboratory of Polymer Science and Technology, College of Materials Science and Engineering, Shenzhen University, Shenzhen 518060, China; 2160120407@email.szu.edu.cn (T.W.); xiaojunyin@szu.edu.cn (X.Y.); pancj@szu.edu.cn (C.P.); dqliu@szu.edu.cn (D.L.); zhouxiaoyan16@163.com (X.Z.); 2 College of Chemistry and Chemical Engineering, Shenzhen University, Shenzhen 518060, China; 3 Department of Applied Biology and Chemical Technology, The Hong Kong Polytechnic University, Hung Hom, Hong Kong, China

**Keywords:** thermoelectric, platinum acetylides, composite films, adhesivity, carbon nanotubes

## Abstract

Single-walled carbon nanotubes (SWCNTs) incorporated with π-conjugated polymers, have proven to be an effective approach in the production of advanced thermoelectric composites. However, the studied polymers are mainly limited to scanty conventional conductive polymers, and their performances still remain to be improved. Herein, a new planar moiety of platinum acetylide in the π-conjugated system is introduced to enhance the intermolecular interaction with the SWCNTs via π–π and *d*–π interactions, which is crucial in regulating the thermoelectric performances of SWCNT-based composites. As expected, SWCNT composites based on the platinum acetylides embedded polymers displayed a higher power factor (130.7 ± 3.8 μW·m^−1^·K^−2^) at ambient temperature than those without platinum acetylides (59.5 ± 0.7 μW·m^−1^·K^−2^) under the same conditions. Moreover, the strong interactions between the platinum acetylide-based polymers and the SWCNTs are confirmed by scanning electron microscopy (SEM) and transmission electron microscopy (TEM) measurements.

## 1. Introduction

Thermoelectric (TE) materials, which can directly convert thermal energy into electrical energy via the mobility of solid internal carriers, have demonstrated a great potential in both power generation and solid-state cooling or heating [1,2,3,4]. As yet, inorganic TE materials, such as PbTe and Bi_2_Te_3_, have been studied extensively, but their high cost, toxicity, brittleness and inelasticity attributes have greatly hindered their applications [5,6,7]. Compared with the inorganics, organics materials possess many unique advantages, such as low thermal conductivity, low cost, light-weight, flexibility and adjustable molecular structure, rendering them as a fascinating candidate for TE application [8,9]. Since the conversion efficiency of the TE materials is evaluated by the dimensionless figure of merit, *ZT* = *S*^2^*σT/κ*, where *S* is the Seebeck coefficient (μV·K^−1^), *σ* is the electrical conductivity (S·cm^−1^), *κ* is the thermal conductivity (W·m^−1^·K^−1^), and *T* is the absolute temperature (K), respectively [10,11,12], a high *S* accompanied with a high *σ* and a low *κ* is crucial to achieve a high *ZT* value.

It still remains a massive challenge to optimize the *ZT* values in a unitary system due to the interactions between the *σ*, *S* and *κ*, i.e., since in most cases the increase of *σ* relies on the enhancement of the carrier concentration, which may result in diminution of *S* or raising of *κ* [13,14,15]. To alleviate this issue, a composite system by incorporating two or more components may provide a valid approach to address this concern [16,17,18].

Generally, π-conjugated polymers are regarded as favorable organic TE materials due to the potentially high *S* and low *κ*, but the intrinsically low *σ* is far from satisfactory [19,20]; on the other hand, single-walled carbon nanotubes (SWCNTs) have prominent high *σ* and mechanical robustness, but low *S* and high *κ*, which render them as perfect counterparts to organic TE materials. In this regard, SWCNTs incorporated with π-conjugated polymers have been effectively applied to produce TE composites. For example, Yao et al. reported SWCNT/polyaniline (PANI) nanocomposites with both improved σ and *S* compared to the individual PANI, with a maximum power factor (PF = *S*^2^*σ*) up to 20 μW·m^−1^·K^−1^, which is more than 2 orders of magnitude higher than the pure PANI [21]; Chen et al. tried the polypyrrole (PPy) nanowire/SWCNT composite films, and achieved a maximum PF of 21.7 ± 0.8 μW·m^−1^·K^−1^, which is 998 times higher than that of the pure PPy nanowires [22]. Despite numerous polymer/SWCNT TE composites have been studied, the available polymers are mainly focused on scanty conventional conductive polymers, such as polypyrrole [23,24,25], poly(3,4-ethylenedioxythiophene) [26,27,28], polythiophene [29,30,31], polyaniline [32,33,34] and their derivatives. In particular, the *S* of the composites declined sharply with the increase in the loading of SWCNTs, which leads to difficulty for TE performance optimization. 

**Scheme 1:**
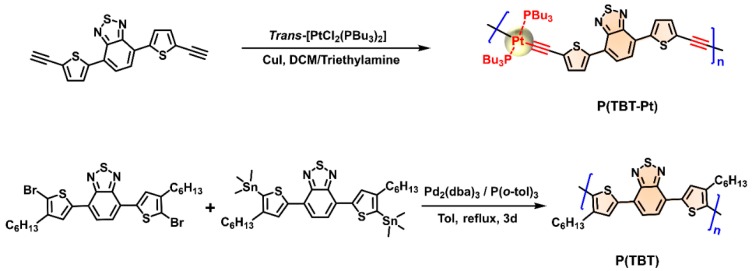
Synthetic routes of the platinum (II) acetylide based copolymer, P(TBT-Pt), and the TBT-based homopolymer with platinum acetylide unit absent in the π-conjugated main chain, namely P(TBT), where TBT is 4,7-di(thiophen-2-yl)benzo[*c*][1,2,5]thiadiazole.

In this work, new polymer 4,7-di(thiophen-2-yl)benzo[*c*][1,2,5]thiadiazole (TBT) and platinum (II) acetylide based copolymer, P(TBT-Pt), was designed (Scheme 1). The incorporation of a planar moiety of platinum acetylide into the π-conjugated system can significantly enhance the intermolecular interaction with the SWCNTs by π–π interactions, while the large steric ligand of *tri*-n-butylphosphine may provide an additional obstacle to restrain the π–π stacking between the backbones of P(TBT-Pt), which is crucial in regulating the performances of the SWCNT-based composites [35,36,37]. Correspondingly, the TBT-based homopolymer with platinum acetylide unit absent in the π-conjugated main chain, namely P(TBT), was synthesized in parallel for comparison (Scheme 1). Remarkably, the *S* of P(TBT-Pt) and SWCNT hybrid films were slightly decreased with the increase of SWCNT (from 53.5 ± 1.3 μV·K^−1^ at 10% SWCNT to 44.3 ± 0.9 μV·K^−1^ at 90% SWCNT), while the *σ* of the composites were gradually enhanced with the increase of SWCNT loading, which enabled a high PF of 130.7 ± 3.8 μW·m^−1^·K^−1^ at ambient temperature. On the contrary, the P(TBT)/SWCNT composites revealed a significant decrease in *S* from 72.4 ± 1.0 μV·K^−1^ (10% SWCNT) to 26.2 ± 0.4 μV·K^−1^ (90% SWCNT), and resulted in an inferior PF of 59.5 ± 0.7 μW·m^−1^·K^−1^. The temperature-dependent TE performance was characterized from r.t. to 400 K, and an optimized PF of 158.6 μW·m^−1^·K^−1^ was achieved for P(TBT-Pt)/SWCNT (1:9), and 121.7 μW·m^−1^·K^−1^ for P(TBT)/SWCNT (1:9). Moreover, the scanning electron microscopy (SEM) and transmission electron microscopy (TEM) measurements revealed a stronger interaction for the P(TBT-Pt)/SWCNT than the P(TBT)/SWCNT, which could also be verified by Raman spectra and X-ray photoelectron spectroscopy studies.

## 2. Experimental

### 2.1. Instrumentation

^1^H NMR and ^13^P NMR spectra were collected by a VNMRS (400MHz) NMR spectrometer (Agilent, Palo Alto, California, CA, USA) in CDCl_3_ with the tetramethylsilane as the internal standard. The average molar mass and distribution were determined by gel permeation chromatography (GPC) (Waters e2695 Separations Module, Waters, Singapore), and the polystyrene was used as a standard. Thermal gravimetric analysis (TGA) was conducted on a TGA-Q50 system (TA Instruments, NewCastle, DE, USA) with a heating rate of 10 °C/min. Fourier transform infrared spectra (FTIR) were collected within the wavenumbers range of 400–4000 cm^−1^ using an FTIR spectrometer (Nicolet 6700, Thermo Fisher Scientific, Waltham, MA, USA). The ultraviolet-visible-near infrared (UV-Vis-NIR) absorption spectra of the polymers were measured in film state using a Lambda 950 (PerkinElmer, Waltham, MA, USA) spectrophotometer. 

The morphologies of both the polymer films and composite films were investigated by scanning electron microscopy (SEM, SU-70, Tokyo, Japan) and transmission electron microscopy (TEM, JEM-1230, JEOL, Tokyo, Japan). Raman spectra were collected within the wavenumbers range from 500 to 3000 cm^−1^ using a Raman spectrometer (Renishaw inVia™ Raman Microscope, London, England), with an excitation wavenumber of 514.5 nm. The electron binding energy was obtained from X-ray photoelectron spectroscopy (XPS, ESCALAB 250Xi, Thermo Fisher Scientific, Waltham, MA, USA). Cyclic voltammetry (CV) of the two polymers was done on a CHI 660E electrochemical workstation (Chenhua Instruments Co., Shanghai, China) in a 0.1 mol·L^−1^ tetrabutylammonium tetrafluoroborate (Bu_4_NBF_4_) acetonitrile solution. Platinum disk, platinum wire and Ag/Ag^+^ were used as the working, counter, and reference electrodes, respectively. The CV samples were prepared by using deposited thin films, which were obtained by drop-casting the polymers solution (chlorobenzene, 20 mg/mL) onto the platinum disk until the chlorobenzene evaporated completely. The scan rate was 0.05 V·s^−1^ under the nitrogen atmosphere. The electrochemical potentials were calibrated by ferrocene (Fc/Fc^+^) and the half potential (E_1/2_) of Fc/Fc^+^ was measured as 0.09 V against Ag/Ag^+^.

### 2.2. Materials Preparation

All commercially available reagents were used directly without further treatment, unless otherwise noted. The single-walled carbon nanotubes (SWCNTs) (diameter: <3 nm, purity: >95.0 wt %), were purchased from XFNANO Materials Technology Co., Ltd., Nanjing, China. Monomers of 4,7-bis(5-bromothiophen-2-yl)benzo[*c*][1,2,5]thiadiazole (1), 4,7-bis(5-bromo-4-hexylthio-phen-2-yl)benzo[*c*][1,2,5]thiadiazole (2) and 4,7-bis(4-hexyl-5-(trimethylstannyl)thiophen-2-yl)benzo[*c*][1,2,5]thiadiazole (3) were purchased from Derthon Optoelectronic Materials Science Technology Co., Ltd., Shenzhen, China. The initial materials of *trans*-[PtCl_2_(PBu_3_)_2_] and 4,7-bis(5-ethynylthiophen-2-yl)benzo[*c*][1,2,5]thiadiazole (4) were prepared according to the literature methods [38,39,40].

#### 2.2.1. Synthesis of the Polymer P(TBT-Pt)

A mixture of 4 (429 mg, 0.83 mmol) and *trans*-[PtCl_2_(PBu_3_)_2_] (557 mg, 0.83 mmol) in the NEt_3_/CH_2_Cl_2_ mixed solution (40 mL, *v*/*v*, 1:1) was stirred at room temperature for 72 h in the presence of CuI (8 mg, 5%) as the catalyst. Then the solution was diluted with chloroform (CHCl_3_) and washed with water. The organic layer was dried over Na_2_SO_4_ and filtered. The solvent was removed by evaporation under reduced pressure, and the remaining solid was dissolved in a small amount of tetrahydrofuran (THF) solution, and then dispersed into acetone. The target polymer was collected by centrifugation of the suspensions (4000 rpm, 30 min). The polymer P(TBT-Pt) was obtained as a dark purple solid in 97% yield (900 mg, 0.81 mmol). ^1^H NMR (400 MHz, CDCl_3_): δ 7.98 (s, 2H), δ 7.73 (s, 2H), δ 6.93–6.92 (m, 2H), δ 2.16 (s, 12H), δ 1.55–1.48 (m, 24H), δ 0.99–0.96 (m, 18H). ^31^P NMR (243 MHz, CDCl_3_): δ 3.47. GPC (THF, polystyrene standards): M_w_ = 37892, M_n_ = 21999, DP = 22, PDI = 1.72. 

#### 2.2.2. Synthesis of Polymer P(TBT)

A mixture of 2 (523 mg, 0.84 mmol) and 3 (500 mg, 0.84 mmol) in 30 mL chlorobenzene solution was stirred at 110 °C for 72 h in the presence of Pd_2_(dba)_3_ (29 mg, 5%) and P(*o*-tol)_3_ (64 mg, 25%). The solution was then diluted with CHCl_3_ and washed with water. The organic layer was dried over Na_2_SO_4_ and filtered. The solvent was removed by evaporation under reduced pressure, and the remaining solid was dissolved within a small amount of THF solution, and then dispersed into acetone. The target polymer was collected by centrifugation of the suspensions (4000 rpm, 30 min). The polymer P(TBT) was obtained as a dark red solid in 86% yield (692 mg, 0.72 mmol). ^1^H NMR (400 MHz, CDCl_3_): δ 8.10 (s, 2H), δ 7.86 (s, 2H), δ 2.73–2.66 (m, 4H), δ 1.73–1.66 (m, 4H), δ 1.33–1.27 (m, 12H), δ 0.88–0.83 (m, 6H). GPC (THF, polystyrene standards): M_w_ = 12,598, M_n_ = 10,412, DP = 21, PDI = 1.21.

### 2.3. Preparation of the Composite Films

**Scheme 2:**
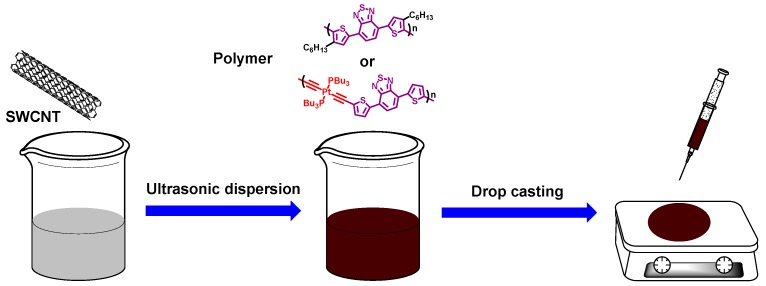
Preparation of P(TBT-Pt)/Single-walled carbon nanotubes (SWCNTs) and P(TBT)/SWCNT composite thin films.

As shown in Scheme 2, SWCNT (10 mg) was dispersed in anhydrous chlorobenzene (10 mL) using a probe sonicator (JY99-IIDN, Scientz, Ningbo, China) under a cooling bath for 30 min. Then an appropriate amount of P(TBT-Pt) or P(TBT) (in chlorobenzene,10 mg/mL) was added into the dispersion of these SWCNTs. Afterwards, the mixture was mildly sonicated for an additional 3 h to ensure the thorough mixing. The P(TBT-Pt)/SWCNTs composition was varied by adding different amounts of P(TBT-Pt). Microscope glass slides were cut into 15 mm × 15 mm pieces, and washed sequentially with deionized (DI) water, acetone, isopropanol and methanol. Finally, the mixture was cast on the cleaned glass substrates under ambient conditions to form composite films with thicknesses of 1.2–2.0 μm.

### 2.4. Density Functional Theory Simulations

In order to explore the highest occupied molecular orbital (HOMO) and lowest unoccupied molecular orbital (LUMO) energy levels and distributions, density functional theory (DFT) simulation was carried out at the B3LYP/6-31G* level by Spartan software (version 2016, Wavefunction Inc., Irvine, CA, USA). Particularly, dimers of the polymers were utilized and the alkyl chains were replaced by methyl groups to simplify the DFT simulation.

### 2.5. Thermoelectric Measurements

The Seebeck coefficients and electrical conductivities of these composite films were tested on an MRS-3M thin film thermoelectric test system (MRS-3, Wuhan Joule Yacht Science and Technology Co., Ltd., Wuhan, China) under vacuum. This MRS-3M thin film thermoelectric test system uses the quasi-dynamic method and the four-wire method to measure the film’s Seebeck coefficients and electrical conductivities, respectively. 

During the quasi-dynamic test, a slight continuous change of temperature difference is applied to both ends of the sample, and the thermoelectric potential change at both ends of the sample is measured. The temperature difference (ΔT) and the thermoelectric potential (ΔV) are both linear, and the slope is the Seebeck coefficient (*S*), which is described as S=−ΔV/ΔT. Meanwhile, electrical conductivities were also collected. The Seebeck coefficient was corrected by nickel standard, and the thermoelectric properties of each SWCNT loading was determined by five parallel samples. The thicknesses of the composite films were collected by high-precision probe profiler (ET-4000M, Kosaka Laboratory Ltd., Tokyo, Japan). 

## 3. Results and Discussion

### 3.1. Material Synthesis and Characterization

The synthetic routes of these two polymers are presented in Scheme 1. The polymer P(TBT) was obtained from aryl halide, and the corresponding organotin precursors by the typical Stille coupling reaction with a high yield over 86%. For another polymer, the platinum acetylides embedded polymer of P(TBT-Pt) was synthesized by the CuI/Pd(PPh_3_)_4_-catalyzed coupling reaction, in the presence of terminal alkynes precursor and *trans*-[PtCl_2_(PBu_3_)], in a good yield over 97%. Both of the two polymers were well characterized by ^1^H NMR spectroscopy and gel permeation chromatography (GPC) analysis (Figure S1). Herein, in order to exclude the influence of the degree of polymerization (DP) on the thermoelectric properties, both of P(TBT-Pt) and P(TBT) were deliberately synthesized with almost the same DP; i.e., DP = 22 for P(TBT-Pt), and DP = 21 for P(TBT) (Figure S1). Noticeably, all these polymers exhibited favorable solubility in common organic solvents, such as chloroform, chlorobenzene, and tetrahydrofuran, which insure a considerable film forming procedure from drop-casting. The typically C≡C stretching vibration of P(TBT-Pt) was confirmed with FTIR spectrum (2081 cm^−^^1^, Figure S3). The thermal-decomposition temperatures (*T_d_*, corresponding to 5% weight loss) of these two polymers were estimated to be 586 K for P(TBT) and 600 K for P(TBT-Pt) (Figure S2).

### 3.2. DFT Simulation, Electrochemical and Photophysical Properties

**Figure 1:**
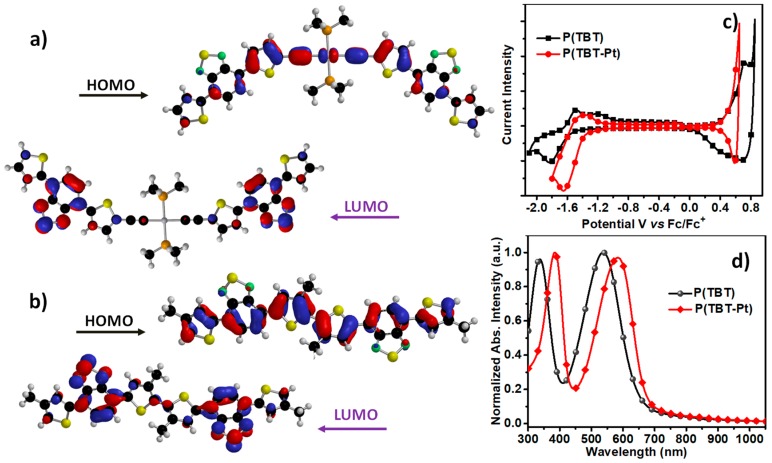
Highest occupied molecular orbital (HOMO) and lowest unoccupied molecular orbital (LUMO) distribution of (**a**) the P(TBT-Pt) and (**b**) the P(TBT) dimers (with alkyl side chain being simplified to methyl). The (**c**) cyclic voltammetry (CV) curves and (**d**) UV-Vis-NIR absorption spectra of these two polymers.

To explore the structural differences between these two polymers on their TE performances, elementary properties including HOMO/LUMO energy levels and distributions, as well as optical bandgaps (*E*_g_), were discussed in priority. As shown in Figure 1a, the HOMOs of P(TBT-Pt) were mainly located at the central platinum acetylides and adjacent thiophene units, while the P(TBT) demonstrated almost uniformly distributed HOMOs along the π-skeleton (Figure 1b). The LUMOs of P(TBT-Pt) were largely localized in the diazosulfide moieties, resulting in segregated HOMO/LUMO distribution and an enhanced intramolecular charge transfer (ICT) state [41]. The electrochemical behaviors of the polymers were investigated by cyclic voltammetry (CV) in acetonitrile, and the energy levels were calculated according to the equation of *E*_energy level_ = −[4.8 + (*E*_onset_ − *E*_1/2(Fc/Fc+)_)] [42,43]. As Figure 1c demonstrated, the onset potentials of the oxidation process were 0.51 eV for P(TBT-Pt) and 0.47 for P(TBT), corresponding to the HOMO levels of −5.31 and −5.27 eV, respectively. In the same way, their LUMO levels could be inferred from the reduction process with −3.42 and −3.25 eV for P(TBT-Pt) and P(TBT), respectively. In comparison with P(TBT), the absorption peaks of P(TBT-Pt) exhibited obvious red-shifts which could be ascribed to the enhanced ICT property of the latter (Figure 1d). Remarkably, the variation of energy levels and *E*_g_s may profoundly influence the activation energies of the charge carriers, and thus determine the TE performances.

### 3.3. Thermoelectric Properties

**Figure 2:**
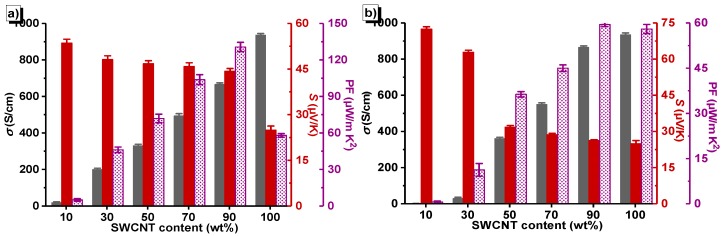
The histogram of *σ*, *S* and PF of (**a**) P(TBT-Pt) and (**b**) P(TBT) *versus* different SWCNT loading.

To evaluate the TE properties of these polymers, composite hybrid films with different SWCNT loadings were prepared, and their *S*, *σ* and PF at room temperature were measured. Herein, for pure P(TBT-Pt) and P(TBT) films, no valid thermoelectric data were obtained due to the intrinsic low *σ*. As shown in Figure 2a, the *σ* of the P(TBT-Pt)/SWCNT composite films was gradually enhanced with the increase of SWCNT loading, which demonstrated a typically SWCNT-assisted conductivity that the increment of SWCNT may facilitate to weave continuous conductive networks. Interestingly, the *S* of P(TBT-Pt)-based composites exhibited negligible decrease with the increase of SWCNT (from 53.5 ± 1.3 μV·K^−1^ at 10% SWCNT to 44.3 ± 0.9 μV·K^−1^ at 90% SWCNT). On the other hand, the *S* of P(TBT)-based composites was sharply decreased from 72.4 ± 1.0 μV·K^−1^ (10% SWCNT) to 26.2 ± 0.4 μV·K^−1^ (90% SWCNT) (Figure 1b). Noticeably, the initially higher *S* of the P(TBT) than the P(TBT-Pt) may be due to the wider band gap of the former than the latter, which results in higher activation energies to prevent the passage of the low-energy carriers. However, the increase of carrier concentration along with the SWCNT loading could diminish the *S* of the composites. Even so, the negligible decrease of *S* with respect to the P(TBT-Pt)-based composites may be due to the embedded platinum acetylides which lead to enhanced π–π interactions between the polymers and the SWCNTs. Herein, the passage of low-energy carriers can be selectively prevented, whereby sandwiching the platinum acetylides between the SWCNTs. Therefore, the optimized PF of P(TBT-Pt) hybrid films (130.7 ± 3.8 μW·m^−1^·K^−1^) was significantly higher than that of the P(TBT)-based films (59.5 ± 0.7 μW·m^−1^·K^−1^) (Table 1).

**Figure 3:**
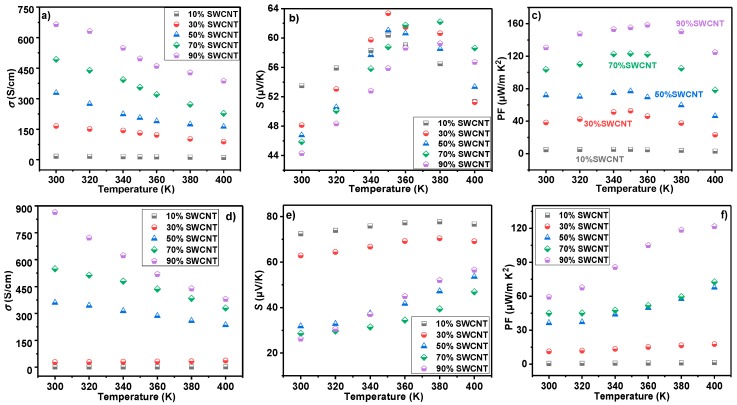
Temperature dependences of the *σ*, *S* and PF, (**a**–**c**) for P(TBT-Pt) and (**d**–**f**) for P(TBT).

To investigate the temperature dependences of the TE properties of these composites, the *S*, *σ* and PF of these samples under variable-temperature model were performed (Figure 3). Unexpectedly, all these composites (at high SWCNT loading) exhibited a metallic or degenerate semiconducting behavior with the increase of temperature (Figure 3a,d), which may presumably be due to the metallicity of the SWCNT [44]. Obviously, all these hybrid films demonstrated an enhanced *S* with the increase of temperature (Figure 3b,e), which could be interpreted in terms of a thermally-assisted hopping mechanism. 

Thus, an optimized PF of 158.6 μW·m^−1^·K^−1^ can be achieved by P(TBT-Pt)/SWCNT (1:9) composite, while an inferior PF of 121.7 μW·m^−1^·K^−1^ was achieved by P(TBT)/SWCNT (1:9) composite (Table S1). Noticeably, the insertion of platinum acetylides among the π-conjugated skeletons may significantly promote the intermolecular interactions between the SWCNT and the P(TBT-Pt) (i.e., pπ–pπ or dπ–pπ interactions), and thus boost the TE performances of the composites. In addition, both P(TBT) and P(TBT-Pt) exhibited high thermal-decomposition temperatures (*T_d_*, corresponding to 5% weight loss) over 573 K, rendering a favorable thermal stability of the composites, such as, both the P(TBT-Pt)/SWCNT (50% wt %) and the P(TBT)/SWCNT (50% wt %) composites showed a high *T_d_* over 573 K (Figure S2).

### 3.4. SEM and TEM Image Studies

**Figure 4:**
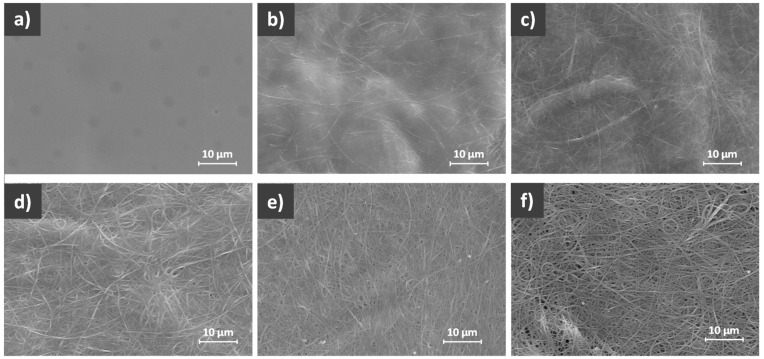
Scanning electron microscopy (SEM) images of the P(TBT-Pt)/SWCNTs composite films with different SWCNT loading, (**a**) 0%, (**b**) 10%, (**c**) 30%, (**d**) 50%, (**e**) 70%, (**f**) 90%.

**Figure 5:**
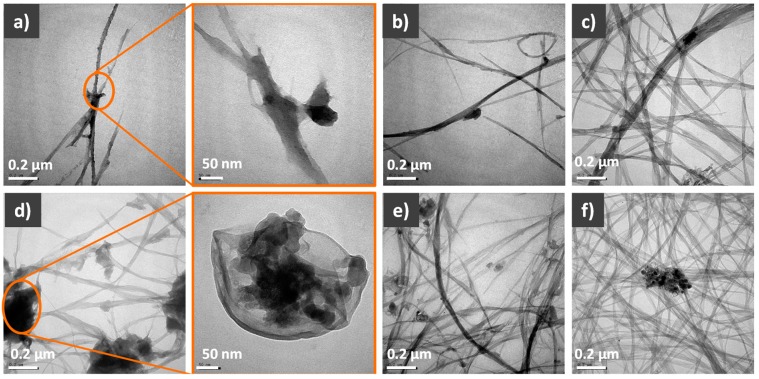
Transmission electron microscopy (TEM) images of the composites for P(TBT-Pt)/SWCNT (**a**) 5:5, (**b**) 3:7, (**c**) 1:9, or P(TBT)/SWCNT (**d**) 5:5, (**e**) 3:7, (**f**) 1:9.

The SEM and TEM measurements were performed to provide a direct insight into the morphologies and interactions between the SWCNTs and the π-conjugated polymers. As depicted in Figure 4, all of these P(TBT-Pt)-based composite films exhibited visually homogeneous surfaces, regardless of any variety of hybrid ratios, which revealed favorable interactions between the SWCNT and the P(TBT-Pt). Noticeably, these platinum acetylides are dispersed well among the carbon nanotube bundles that will be beneficial for the TE performance optimization. On the contrary, P(TBT) without platinum acetylides containing in the π-conjugated backbones exhibited significantly different morphologies. As Figure S4 demonstrated, obvious surface inhomogeneity can be observed with the increase in the SWCNT content, indicating a poor interaction between the SWCNT and the P(TBT). In view of the theoretical simulation results, both P(TBT-Pt) and P(TBT) exhibited a parallel planar molecular structure (Figure 1). Nevertheless, in comparison with P(TBT), the following two aspects may provide an ideal contact between P(TBT-Pt) and SWCNTs: (1) An appropriate steric hindrance from tributylphosphine ligand to restrain the intermolecular aggregates among the P(TBT-Pt) chains, and (2) an additional pπ and dπ orbitals from platinum acetylides to enhance the intermolecular π–π interactions with the SWCNTs. On the other hand, more immediate evidences to elucidate such difference can be observed from the TEM images. As displayed in Figure 5, the P(TBT-Pt) are polymers dispersed well among the SWCNT networks, and in particular, the SWCNTs can be wrapped perfectly by the P(TBT-Pt) chains at high P(TBT-Pt)/SWCNT ratios (Figure 5a). However, obvious aggregation of P(TBT) occurred in the P(TBT)/SWCNT hybrid system due to the interactions between the P(TBT) chains and SWCNTs. 

### 3.5. Raman Spectroscopy

**Figure 6:**
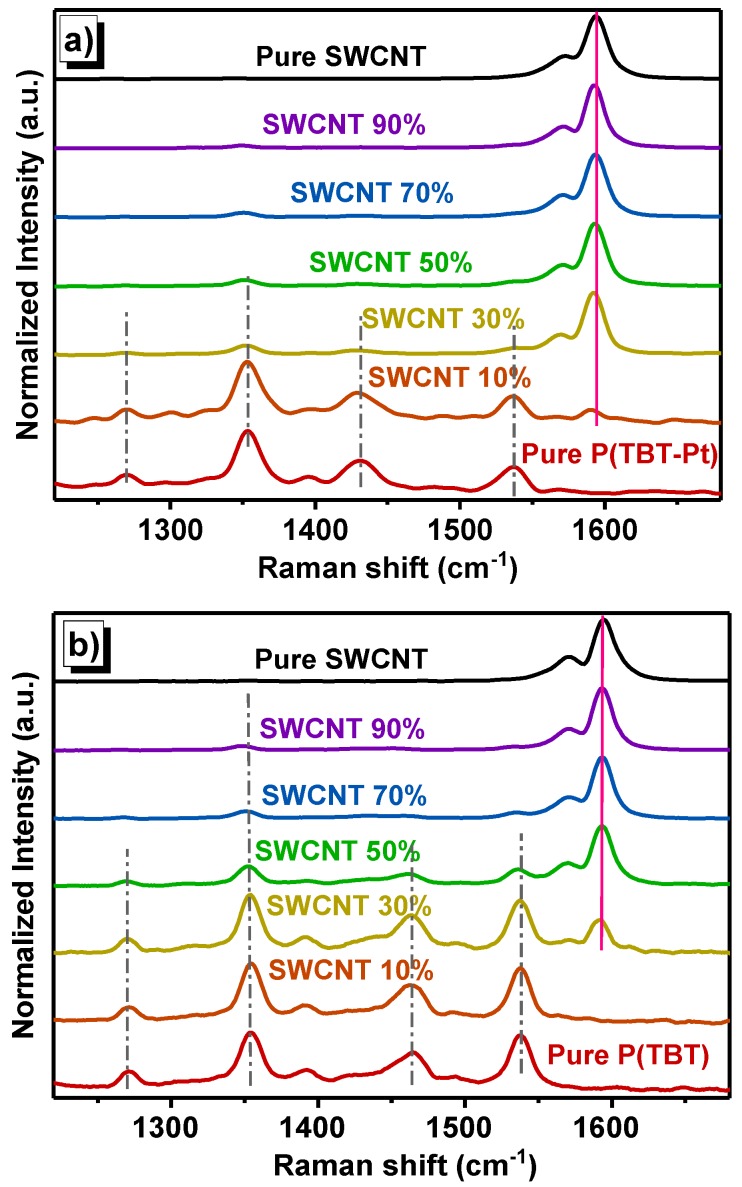
Raman spectra of the (**a**) P(TBT-Pt)/SWCNT and (**b**) P(TBT)/SWCNT composite films.

The Raman spectra of the P(TBT-Pt)/SWCNT and P(TBT)/SWCNT composite films were performed to distinguish the interactions between the polymers and the SWCNTs at the molecular vibrational and rotational level. As revealed in Figure 6, both P(TBT-Pt) and P(TBT) exhibited a typical vibration peak at about 1350 cm^−1^ (assigned to the C–H in-plane stretching vibration of the thiadiazole unit [45]), a peak at around 1430 cm^−1^ (assigned to the symmetrical C=C stretching mode for the thiophene unit [46]), and a peak at nearby 1538 cm^−1^ (assigned to the stretching vibration of benzene ring of the benzothiadiazole unit [47]). For pure SWCNTs, the typical graphite-like G band appeared at around 1591 cm^−1^, while the defect-induced resonant scattering (D band) was shown at about 1346 cm^−1^. Noticeably, all the composite films exhibited insignificant D band signal, revealing that no evident structure defects were introduced after the film-forming procedure. As expected, the characteristic Raman bands of the polymers gradually faded away with the increase of the SWCNT proportion (Figure 6). However, for P(TBT-Pt)/SWCNT composites, the Raman signal of P(TBT-Pt) was almost vanished at a very low SWCNT loading of 30% in contrast to the 70% loading for the P(TBT) system, which may presumably be due to the enhanced π–π interactions between the SWCNT and the P(TBT-Pt). 

Noticeably, the enhanced adhesivity of the polymers towards the SWCNTs can largely suppress the vibrational and rotational of the moieties itself. In addition, all the intrinsic vibrational peaks of P(TBT-Pt) exhibited 3–5 cm^−1^ red-shift in P(TBT-Pt)/SWCNT composites, which can be ascribed to the enhanced π–π interactions between the P(TBT-Pt) and the SWCNTs [48]. However, in comparison with the P(TBT), no obvious peak shifts can be observed in P(TBT)/SWCNT composites.

### 3.6. X-ray Photoelectron Spectroscopy Studies

**Figure 7:**
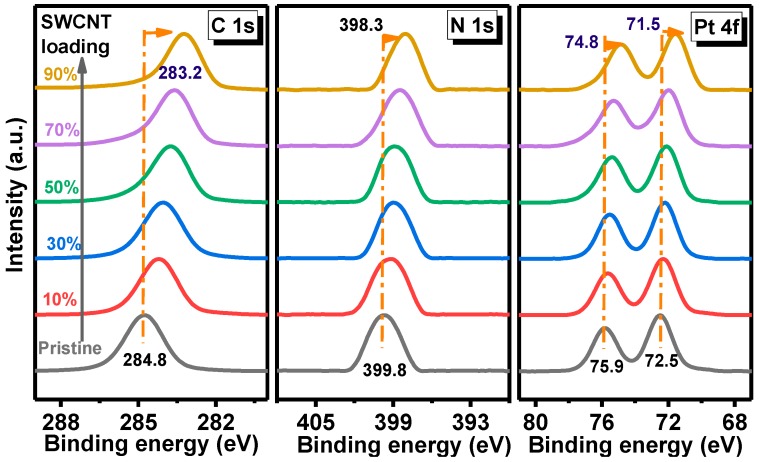
The C 1s, N 1s and Pt 4f spectra of the P(TBT-Pt)/SWCNT composite films.

To further investigate the distinctions of the P(TBT-Pt)/SWCNT and the P(TBT)/SWCNT hybrid systems, the X-ray photoelectron spectroscopy (XPS) analysis was carried out. As shown in Figure 7, the binding energies of the C 1s, N 1s and Pt 4f peaks were significantly shifted with the variation of the P(TBT-Pt)/SWCNT hybrid ratios. Noticeably, the C 1s, N 1s and Pt 4f peaks all gradually shifted to the low binding energy regions along with the increase of SWCNT loading, which indicated a more delocalized electronic surrounding. Based on the aforementioned results, we infer that the shifted binding energies could largely be attributed to the pπ–pπ and dπ–pπ interactions between P(TBT-Pt) and SWCNTs. On the other hand, similar trends can also be observed in the XPS spectra of the P(TBT)/SWCNT composite films (Figure S5). However, in this case, the peaks shifting towards the low binding energy regions may presumably be due to the aggregation-induced π–π stacking among the P(TBT) chains, and partly the pπ-pπ interactions between the P(TBT) and the SWCNTs.

## 4. Conclusions

In conclusion, a novel platinum (II) acetylide polymer of P(TBT-Pt), and a homologue structure of P(TBT) with platinum acetylide units missing in the main chains, were designed and synthesized. In comparison with P(TBT), the HOMO energy levels and optical band gaps of P(TBT-Pt) were only slightly shifted. However, the insertion of platinum acetylides could significantly enhance the adhesivity of the polymers towards SWCNTs, and hence relieve the decrease of *S* with the increase in the loading of SWCNTs. As a consequence, the optimized PFs of the P(TBT-Pt)/SWCNT composites were almost 3 times higher than the P(TBT)/SWCNT composites. The SEM and TEM studies give a direct insight to confirm our assumption, and such results could also be supported by the Raman spectra and XPS measurements. Notably, the insertion of platinum acetylides into the π-conjugated polymers demonstrated in this work suggested an effective approach to alleviate these contradictions between the *σ* and *S* for polymer/SWCNT hybrid TE systems.

## Figures and Tables

**Table 1:** Thermoelectric (TE) parameters of P(TBT-Pt) and P(TBT) hybrid films at room temperature.

SWCNT (wt%) ^(a)^	P(TBT-Pt)/SWCNT			P(TBT)/SWCNT		
	*σ* [S·cm^−1^]	*S* [μV·K^−1^]	PF [μW·m^−1^·K^−1^]	*σ* [S·cm^−1^]	*S* [μV·K^−1^]	PF [μW·m^−1^·K^−1^]
10%	17.2 ± 5.6	53.5 ± 1.3	4.9 ± 1.0	1.2 ± 0.6	72.4 ± 1.0	0.6 ± 0.3
30%	199.0 ± 7.8	48.1 ± 1.2	46.1 ± 2.3	28.4 ± 7.8	62.9 ± 0.8	11.3 ± 2.1
50%	328.8 ± 9.3	46.8 ± 0.9	71.9 ± 3.5	360.0 ± 8.1	31.8 ± 0.7	36.4 ± 0.9
70%	493.1 ± 9.5	45.9 ± 1.1	103.8 ± 4.1	548.9 ± 9.4	28.6 ± 0.6	45.0 ± 1.1
90%	665.8 ± 8.9	44.3 ± 0.9	130.7 ± 3.8	864.4 ± 8.8	26.2 ± 0.4	59.5 ± 0.7
100%	934.7 ± 9.9	24.9 ± 1.4	58.0 ± 1.5			

^**(a)**^ Refer to the mass ratios of SWCNT.

## Supplementary Materials

The following are available online at https://www.mdpi.com/2073-4360/11/4/593/s1.
