# Supplementary material for: Boosting the Adhesivity of π-Conjugated Polymers by Embedding Platinum Acetylides towards High-Performance Thermoelectric Composites
Source: Polymers (Basel). 2019 Apr 1;11(4):593. doi: 10.3390/polym11040593 (PMC6523095; doi:10.3390/polym11040593)
Supplement: Supplementary file 1 [file polymers-11-00593-s001.pdf]

Supporting information for:

Boosting the adhesivity of π-conjugated polymers by embedding platinum acetylides towards high-performance thermoelectric composites

Tao Wan [1], Xiaojun Yin [1], Chengjun Pan [1], Danqing Liu [1], Xiaoyan Zhou [1], Chunmei Gao [2,]*, Wai-Yeung Wong [1,3,]* and Lei Wang [1,]*

[1] Shenzhen Key Laboratory of Polymer Science and Technology, College of Materials Science and Engineering, Shenzhen University, Shenzhen 518060, China; 2160120407@email.szu.edu.cn (T.W.), xiaojunyin@szu.edu.cn (X.Y.), pancj@szu.edu.cn (C.P.), dqliu@szu.edu.cn (D.L.), zhouxiaoyan16@163.com (X.Z.)

[2] College of Chemistry and Chemical Engineering, Shenzhen University, Shenzhen 518060, PR China

[3] Department of Applied Biology and Chemical Technology, The Hong Kong Polytechnic University, Hung Hom, Hong Kong, China

* Correspondence: gaocm@szu.edu.cn (C.G.); wai-yeung.wong@polyu.edu.hk (W.-Y.W.); wl@szu.edu.cn (L.W.)

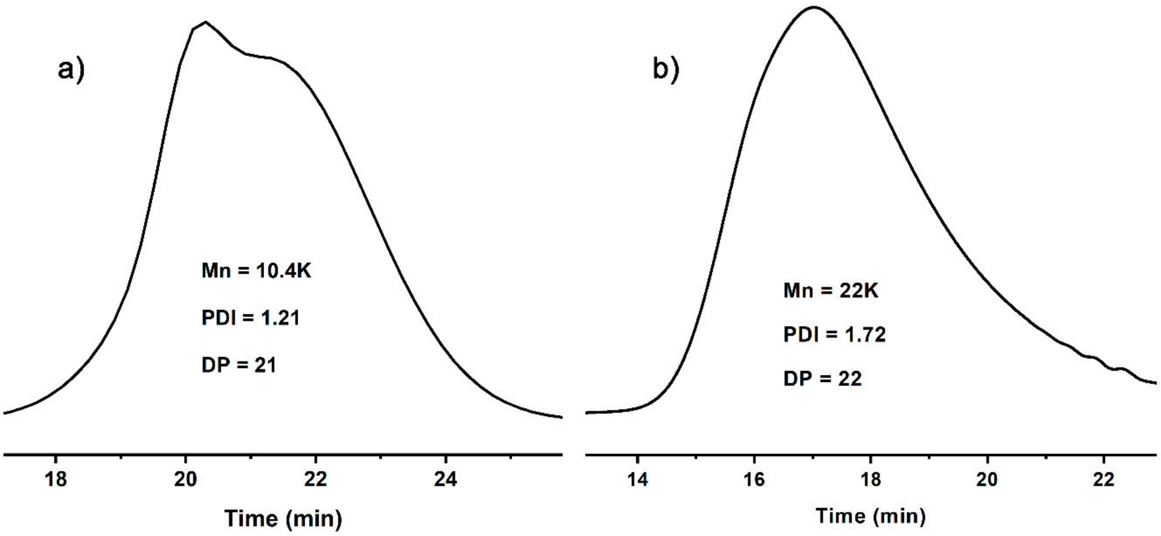

Figure S1. Gel permeation chromatography (GPC) curves of the polymers the TBT-based homo-polymer with platinum acetylide unit absent in the π-conjugated main chain, namely P(TBT) and platinum (II) acetylide based copolymer, P(TBT-Pt), where TBT is 4,7-di(thiophen-2-yl)benzo[*c*]-[1,2,5]thiadiazole.

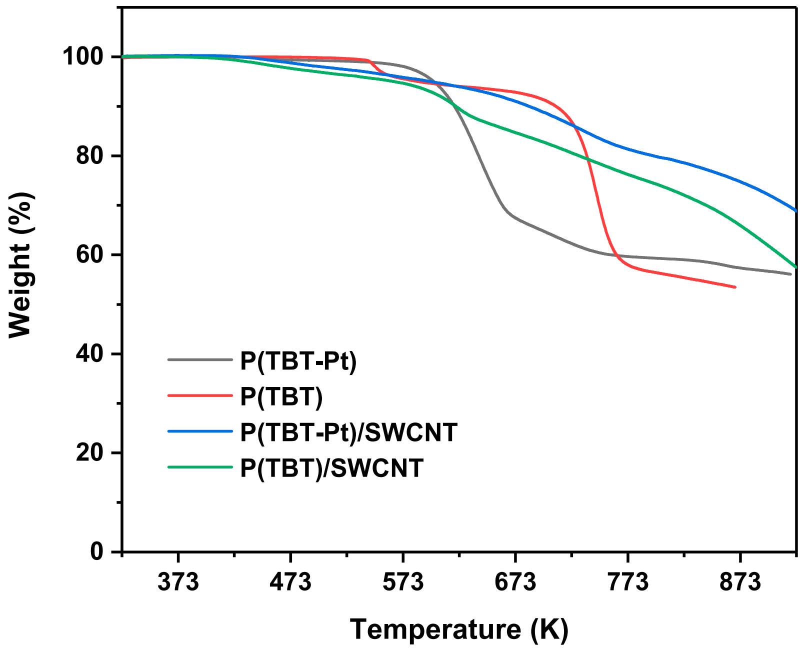

Figure S2. Thermal gravimetric analysis (TGA) curves of the P(TBT-Pt), P(TBT), P(TBT-Pt)/SWCNT (1:1, wt %) and P(TBT)/SWCNT (1:1, wt %) samples.

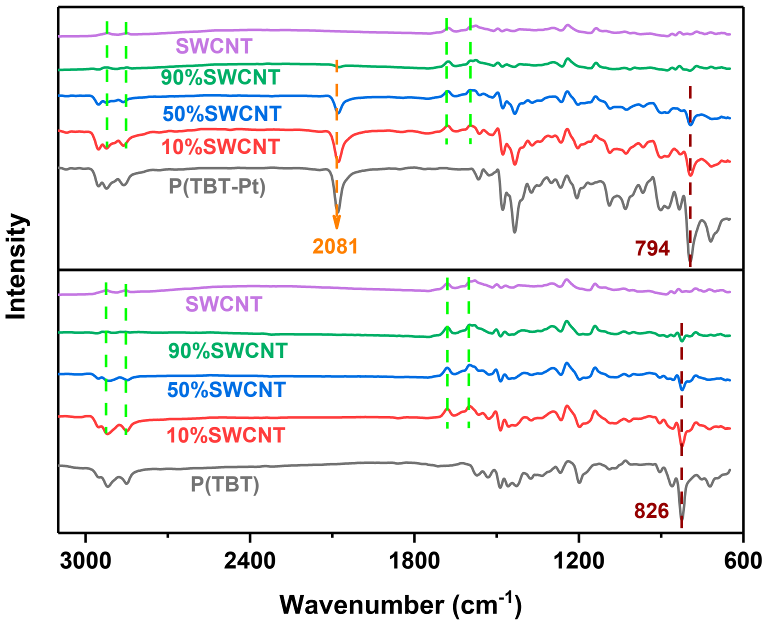

Figure S3. FTIR curves of the P(TBT-Pt)/SWCNT and P(TBT)/SWCNT hybrid films.

As shown in Figure S3, in comparison with the pristine P(TBT-Pt) film, the characteristic absorption peaks of the platinum acetylides (at around 2081 cm^{-1}) in P(TBT-Pt)/SWCNT composites exhibited 2 ~ 4 cm^{-1} red – shift, which indicated enhanced π-π interactions between the P(TBT-Pt) and the SWCNTs.

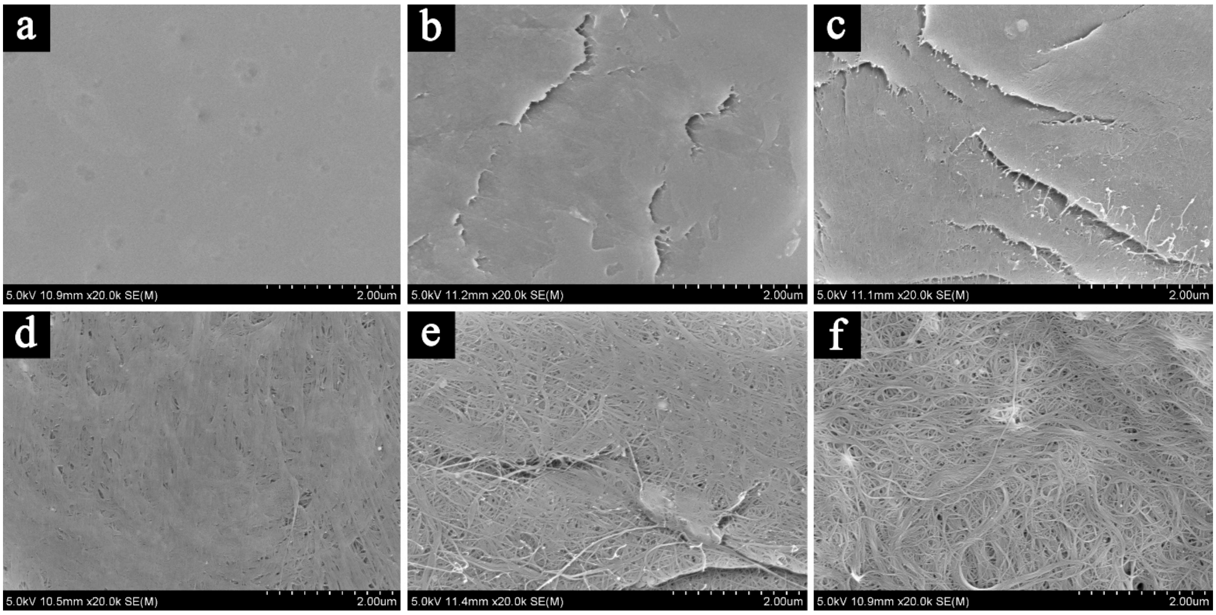

Figure S4. Scanning electron microscopy (SEM) images of the P(TBT)/SWCNTs composite films with different SWCNT loding, a) 0%, b) 10%, c) 30%, d) 50%, e) 70%, f) 90%.

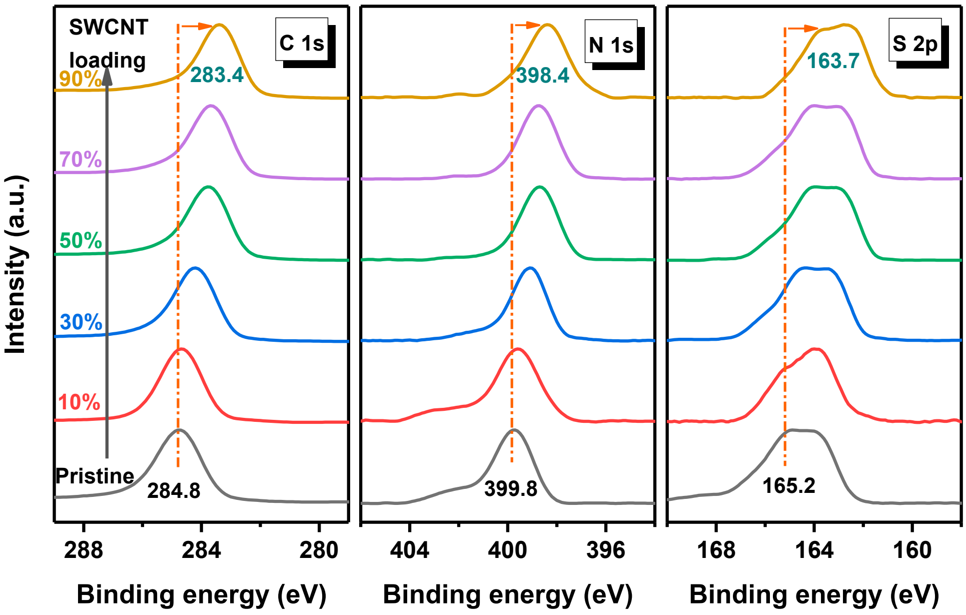

Figure S5. The C 1s, N 1s and S 2p spectra of the P(TBT)/SWCNT composite films.

Table S1. Key thermoelectric parameters of the P(TBT-Pt)/SWCNT and P(TBT)/SWCNT composite films under different temperature (from r.t. to 400 K).

Composites	σ_{max} [S·cm^{-1}]	S_{max} [μV·K^{-1}]	PF$_{max}$ [μW·m^{-1}·K^{-2}]
**P(TBT-Pt) /SWCNT**	674.7	63.4	158.6
**P(TBT) /SWCNT**	873.2	77.7	121.7
